# Assessing the impact of corn variety and Texas terroir on flavor and alcohol yield in new-make bourbon whiskey

**DOI:** 10.1371/journal.pone.0220787

**Published:** 2019-08-08

**Authors:** Robert J. Arnold, Alejandra Ochoa, Chris R. Kerth, Rhonda K. Miller, Seth C. Murray

**Affiliations:** 1 Department of Soil and Crop Sciences, Texas A&M University, College Station, Texas, United States of America; 2 Firestone & Robertson Distilling Co., Fort Worth, Texas, United States of America; 3 Department of Animal Science, Texas A&M University, College Station, Texas, United States of America; Colorado State University, UNITED STATES

## Abstract

The whiskey industry is dominated by whiskey styles with recipes that contain corn as the primary grain. However, little research has been conducted to investigate whiskey specific distinctions arising from different corn varieties and growing environments (i.e. terroir). Further, no studies have investigated the aroma or flavor impacts of different varieties and terroirs. Here, three different commodity yellow dent hybrid corn varieties were grown on different farms in Texas, spanning from the Texas Panhandle to the Mexico-United States border. Using novel small-batch mashing techniques, a newly developed new-make (i.e. unaged whiskey,immediate by-product of distillation) bourbon sensory lexicon, a trained sensory panel, high-performance liquid chromatography, and gas chromatography-mass spectrometry/olfactometry (GC-MS/O), we report for the first time a method for evaluating sample effects on alcohol yield and flavor in new-make bourbon whiskey. We discover that variety, terroir and their interactions, previously ignored, can substantially affect valuable sensory aspects of whiskey, suggesting the importance of scientifically evaluating corn genetics and agronomy for developing better whiskey. Excitingly, our data suggest milled corn with higher levels of benzadehyde, readily measured by GC-MS/O, correlates with improved sensory aspects of distillate, which must be expensively evaluated using a trained human sensory panel.

## Introduction

The United States (US) whiskey industry is dominated by whiskey styles that by law must contain corn (*Zea mays* L.; commonly maize in much of the world) as the main fermentable substrate, or that by choice use corn as a substantial secondary ingredient. “Bourbon whiskey” (or simply “bourbon”), per the Standards of Identity for Distilled Spirits (Title 27, Part 5, Subpart C of the US Code of Federal Regulations), must contain at least 51% corn. However, most “bourbon” brands utilize 70–80% corn. “Corn whiskey”—a much less popular yet still important style—must contain at least 80% corn, with the barrel maturation process differentiating “bourbon” and “corn whiskey”. “Rye whiskey” was the most popular style during in the US throughout the 18^th^ century, and it has seen a recent resurgence—largely due to the recent rise in Prohibition-era cocktails, which often utilized rye whiskey—after almost going extinct. This style must contain at least 51% rye, and many “rye whiskey” brands do indeed utilize near the minimum rye requirement, with corn making up anywhere from 30 to 40% of the recipe. “Wheat whiskey” follows the same trend as rye, in that wheat must be the majority grain, but corn is still present at a fairly high percentage.

Corn is a vital ingredient in many of the US’s most popular whiskeys, and the Canadian whiskey industry is similar in this aspect. Even though rye is often championed, 90% of the grain used by the Canadian whiskey industry is corn [1]. Being that corn is a grain native to North America, domesticated in Mexico [2,3], it is fitting that both the US and Canadian whiskey industries rely primarily on it as the fermentable substrate. In Scotland and Ireland, two of the largest national producers in the whiskey industry, barley grows favorably. Consequently, raw barley is the dominant grain in the Irish style known as “pot still whiskey”; and barley’s downstream derivative barley malt is the dominant grain for Scotch and Irish “malt whiskeys”. However, corn still has a place in these industries. Scotch and Irish *“*grain whiskeys”, which are the main component styles used to create Scotch and Irish “blended whiskeys”, were previously made primarily with corn. In the 1980s, wheat replaced corn in this facet [4]. However, the North British Distillery Company Ltd in Scotland—whose whiskey product is the main component of Johnnie Walker Scotch blended whiskeys (top selling Scotch whiskey worldwide) and Famous Grouse (top selling whiskey in Scotland, and top 10 worldwide)—still utilizes corn as their base ingredient (www.thenorthbritish.co.uk [obtained: June 2018]). Also, the Irish “blended whiskey” Jameson (top selling Irish whiskey worldwide) uses corn as the base for its grain whiskey component (www.jamesonwhiskey.com [obtained: June 2018]). Ultimately, although it might only be marketed in “bourbon” and “corn whiskey”, corn is one of the most prevalent grains in international whiskey production.

Although corn is such an important ingredient in whiskey production, there are few previous reports on how genetics (i.e. variety) and environmental factors (soil conditions, climate, topography, agronomic management, seasonal fluctuations; i.e. terroir) of corn impact alcohol yield and flavor in whiskey. An extensive literature search only resulted in the following reports, where [5] focused on agronomic yield relevant to whiskey distillation; and [6,7,8,9] focused on alcohol yield. To our knowledge, no studies have yet been conducted that investigate how corn variety and terroir impact whiskey flavor. In contrast, barley (and wheat, to a lesser extent) has been a more frequent subject of research, often with support from the Scotch whiskey industry (for a review on barley: [10]; for a review on wheat: [11]). Many of the past barley and wheat studies have focused on how variety and terroir impact agronomic yield. There are a few examples in the literature where alcohol yield was investigated [4,12,13,14,15,16] but none that we know of that have addressed flavor (although [17,18,19,20] did address flavor in regards to beer that was to be consumed before distillation). Further, there are some barley and wheat cultivars that were developed for (either solely or partly) the whiskey industry [10,11]. To date, there are no corn cultivars that have been developed or highlighted for whiskey production; however, the craft distilling movement has revitalized the use open-pollinated heirloom variesties, although this has not been based on science, these heirloom cultivars were not bred solely for alcoholic beverages.

Current protocol among nearly all large-scale bourbon distilleries is to utilize commodity yellow dent hybrid corn [1], which is commonly referred to as field corn. While large-scale distillers will specify a certain grade (at least #2 food grade in the US, which is a grade that requires certain quality standards set buy the United States Department of Agriculture) to ensure acceptable test weight, moisture level, foreign material, and broken/damaged kernels, they will not request a certain variety or a certain terroir. This is in part due to the fact that the logistics of large grain elevators—the main suppliers of grain for large-scale whiskey distilleries—do not readily allow one variety or terroir to be separated and stored until requested [21]. It is also, as previously explained, because a lack of scientific information on end-use differences of corn sources. Comparing this disregard for variety and terroir consideration to the wine industry, it would be analogous to winemakers deciding to make a red wine, and instead of requesting or growing a certain red grape cultivar (e.g. merlot, syrah, pinot noir, etc) and/or terroir (e.g. Napa, Bordeaux, Sonoma, etc.), they would only concern themselves with the color of the grape (red) and some general (but not flavor related) quality specifications. Winemakers, of course, do concern themselves with grape variety and terroir, and they label their wines accordingly. The diversity of flavors among wine grape cultivars is extensive, and therefore many wines are categorized and labeled as a varietal based on their grape cultivar (e.g. merlot). Those that are not labeled as a varietal are usually labeled by their terroir (e.g. Napa Valley, and perhaps even more specific, such as the St. Helena appellation within Napa Valley). And some wines are labeled by both the varietal and the terroir (e.g. Cabernet sauvignon from Napa Valley).

Even if whiskey distilleries did wish to utilize specific corn varieties from specific terroirs in an effort to achieve greater and more consistent alcohol yield and flavor, there has been no reported scientific evidence that the effort would produce desirable or meaningful results. Moreover, the scientific literature on the reduced genetic diversity of modern corn varieties might suggest the opposite, as they would not be expectedto harbor enough genetic variation for distinctions caused by variety and terroir to be realized. As desired agronomic performance traits—such as yield—were pursued in corn, the genetic variability of the species declined [22]. Reports indicate that the majority of recently developed corn inbred lines utilized in American breeding programs are products of a small, stratified, and closed germplasm base [23,24,25]. Additionally, US Corn Belt germplasm can be traced to a narrow range of populations from only two races—the Northern Flint and the Southern Dent [26,27].

Alcohol yield and flavor are quantitative traits [28,29], with many different genes and environmental stimuli influencing the final phenotype. Alcohol yield is primarily correlated with grain starch concentration, the starch composition (i.e. ratio and composition of amylose and amylopectin), and the starch’s propensity to by hydrolyzed by amylases into sugar during mashing [16]. Grain-derived flavors in whiskey can be introduced through multiple pathways. Different sugar, amino acid, and nutrient concentrations and compositions will impact the production of flavor compounds (referred to as *congeners* in the distilled spirits industry) by yeast during fermentation. Also, grain-derived compounds can undergo reactions (such as the Maillard reaction and Strecker degradation) during whiskey processing, which will ultimately deliver flavor compounds to whiskey. Lastly, secondary metabolites (such as pyrrolines, thiazolines, sulfides, lactones, esters, ketones, aldehyhdes, organic acids, indols, and other phytochemicals [30,31]) produced by grain can potentially impact whiskey flavor directly.

If genetic diversity is limited in the relevant pathways among modern yellow dent hybrids, then variety and terroir might not greatly influence alcohol yield and flavor in commodity yellow dent hybrid corn. Conversely, if there is still sufficient genetic diversity among these varieties, then both variety and terroir could have an impact on alcohol yield and flavor, as the relevant genes and how they respond to environmental stimuli would vary. Furthermore, it would be logical that the greater genetic diversity among varieties—for instance, from the other nearly 100 recognized races of corn that exist throughout the Americas [32] or 20,739 accessions in the USDA-ARS National Germplasm Repository [33]—might contain novel flavor profiles far beyond what is currently available in commodity yellow dent hybrid corn.

Ultimately, a specific variety of corn grown in a specific terroir will increase cost and require additional logistics if a distillery identified and wished to utilize it. It would require a distillery to identify a farmer(s) and identity preserve a silo(s), be it one that they build and operate, or one that is managed by a grain elevator. To justify such an endeavor, there must be ample evidence to show that corn variety and terroir can impact alcohol yield and whiskey flavor. This report investigates the variation in alcohol yield and whiskey flavor among three commodity yellow dent hybrids (commercially available varieties in 2016) grown in three to four different experimental field plots in Texas. Only terroirs within Texas (the 11th largest producer of corn in the U.S., USDA-NASS) were considered for this research because of the funder’s interest in sourcing local, Texas-grown corn. However, four very different environments across different regions within Texas were chosen (Table 1).

**Table 1:** Characteristics of different growing locations (i.e. terroirs).

Farm Operation	County	Extension Districts	Soil Type	Planting date	Harvesting date	Plants hectare^-1^	Irrigation	RW	Crop Rotation
Texas AgriLife Extension, Port Lavaca	Calhoun	Coastal bend	Livia silt loam	2/26/2016	8/3/2016	53,987	Dryland	38in	Grain sorghum
Rio Farms, Monte Alto	Hidalgo	South	Raymond-ville clay loam	2/18/2016	7/21/2016	57,027	Three times	30 in	Soybeans
Sawyer Farms	Hill	Central	Houston black clay	Middle February	Middle August	64,218	Dryland	76.2	Wheat
Texas AgriLife Extension	Hansford	Panhandle	Perryton silty clay	5/11/2016	10/11/2016	75,012	Yes	30in	Soybeans

Ppop = Average plant population (Ppop) per hectare. RW = average row width in centimeters between rows. Sawyer Farms is the only commercial grower, with other locations being sites of the Texas A&M (TAMU) Corn Variety Testing Program [34].

The goal of this study was to understand the extent to which variety, terroir, and their interaction can impact alcohol yield and flavor across a wide range of commodity yellow dent varieties and terroirs. This would be infeasible to evaluate at a distillery scale so a repeatable small batch evaluation prodcedure first needed to be developed. Variety, terroir, and the interaction of these factors were treated as random effects so that the results can be extrapolated to more situations than just the three varieties and four terroirs considered here.

## Materials and methods

### Mash, beer, and new-make bourbon production and analyses

New-make samples (i.e. unaged whiskey that is the immediate by-product of distillation) were produced from three varieties of commodity yellow dent hybrid corn obtained from the Texas A&M Corn Variety Testing Program and also from one commercial grower. The three varieties (D57VP51—Dyna-Gro; 2C797—Mycogen Seed; REV25BHR26—Terral Seed) were grown in three different locations in 2016 (Texas AgriLife Extension, Calhoun County, Texas; Rio Farms, Hidalgo County, Texas; Sawyer Farms, Hill County, Texas); an additional location (Texas AgriLife Extension, Hansford County, Texas) was selected to grow one of the varieties (REV25BHR26—Terral Seed). The four terroirs were chosen in an attempt to highlight the diversity of environments in Texas, all within different districts of the Texas A&M AgriLife Extension Service, consisting of varying soil types and agronomic techniques (Table 1).

For the lab-scale milling, mashing, fermentation, and distillation processes, methods were aligned with a laboratory procedure previously developed by the Scotch Whisky Research Institute (SWRI), known to produce a new-make spirit that is comparable to that produced via industrial instrumentation and processes [35,6,36]. Where SWRI methods were created to mimic typical Scotch whisky grain distillery operations, our methods were adapted to more closely simulate typical bourbon whiskey distillery operations.

For processing each batch, whole corn kernel samples were initially sieved through a 0.48 cm round commodity hand sieve (Seedburo Equipment Company) to remove broken kernels. Foreign material and heat-damaged kernels were manually removed via inspection against white paper. The remaining kernels were then milled using a Victoria Plate Mill, and then sieved 3X through a 2000 micrometer screen to ensure that the milled grain was fine and consistent from batch-to-batch. A 3 L beaker was filled with 1750 g of carbon-filtered municipal water. A mechanical mixer (100W-LAB-SM, Gizmo Supply Co.) was used for agitation, and the temperature of the water was brought to 65°C using a 120V hot plate with infinite heat controls (CSR-3T, Cadco) set to medium. Then 448 g of milled corn and 2 mL of high-temperature alpha amylase (AHA-400, FermSolutions Inc.) were added to the beaker. A cover slip that still allowed the mechanical mixer to operate was placed on top of the beaker to prevent excessive evaporation. The temperature of the mash was brought to 85^°^C and held for 1.5 h. After incubation, an ice bath was used to indirectly cool the temperature of the mash to 32^°^C. Once 32^°^C was achieved, 1.5 mL of glucoamylase (GA-150, FermSolutions Inc.) was added. Immediately after, 0.26 g of active dry yeast (Species: *Saccharomyces cerevisiae*; Strain: RHB-422, F&R Distilling Co.’s proprietary strain) was added. The same strain was used for all batches, and the concentration of yeast used was based on standard inoculation rates for the whiskey industry, ensuring the role of other microbial organisms was minimal. The mash was further cooled to 24^°^C using an indirect ice bath and mixed for an additional 10 min. Using aseptic techniques, pH was recorded with a digital pH meter (pH 220C, EXTECH) and specific gravity was recorded using a digital density meter (SNAP 50 density meter, Anton Paar). Further, a 25 mL sample was removed and stored at -20^°^C for high performance liquid chromatography (HPLC) processing. Mixing was then halted, the mash was transferred to a 2.7 L Fernback flask that had been sanitized with Star-San (phosphoric acid based, no rinse sanitizer), and the flask was covered with flame sterilized aluminum foil. Fermentation proceeded for 120 h at room temperature, with pH and specific gravity recorded twice during fermentation, and also at the end of fermentation. Further, 25 mL samples were removed at the same time points and stored at -20^°^C for HPLC processing. Measurements for all 30 treatments were only recorded for Day 0 and Day 5. The treatments recorded for Day 1 (n = 17), Day 3 (n = 15), and Day 4 (n = 27) were chosen at random (Day 2 is not shown due to insufficient data). Three Day 5 outliers were identified based on discrepancies between alcohol yield and ethanol concentration. These outliers were removed from a portion of the analyses. The fermented mash, now called “beer”, was frozen at -20^°^C.

Specific gravity, a measure of density, provides an estimate of fermentable substrate (monosaccharides, disaccharaides, and trisaccharides) and unfermentable substrate (dextrin and starch) yielded via the mashing process (Day 0), the level of attenuation (i.e. the conversion of sugars into alcohol and carbon dioxide by yeast) throughout fermentation (Day 1–4), and the level of attenuation at the end of fermentation (Day 5). The specific gravity (or other corresponding measures of density, such as brix and plato) is one of the most common measurements taken in a distillery, and it is especially important to measure after mashing and during fermentation, as it provides quick and robust insight into process efficiencies. However, specific gravity is ultimately tied to soluble dextrins and sugars, which is why we also conducted follow-up HPLC analyses to quantify these compounds individually.

Beer was rapidly thawed, and 1.65 L was added to the stripping still, which was a stainless steel still with an air fan cooled condenser and an electric, indirect heating element (Air Still, Still Spirits). Distillation proceeded until 550 mL of distillate (termed “low-wines”) was collected in a grade A volumetric flask. The alcohol concentration by volume of the low-wines was measured using a density meter (DMA 5000 M, Anton Paar). Using weight, low-wines were diluted to the desired alcohol concentration with the addition of water. The spirit still, which was a copper alembic style still with a worm coil condenser and no innate heating element (heat was be supplied using the Cadco CSR-3T 120V hot plate with infinite heat controls and set to medium for the spirit run), was charged with 500 mL of low-wines. The condenser was filled with ice water. Distillation commenced, and the first 25 mL of distillate (termed the “heads”) was collected using a grade A volumetric flask. Using a different grade A volumetric flask, the next 100 mL of distillate (termed the “hearts”) was then collected. The condenser was monitored to ensure the temperature of the distillate was consistent from batch-to-batch. The hearts distillate was then stored in Boston round glass bottles with inert caps at room temperature until further processing.

Both stills were cleaned throughout the experiment according to the following methods in order to ensure that the organic residue was not carried-over from batch-to-batch, as well as to ensure that the impact of copper would be consistent from batch-to-batch. These methods were also developed with guidance from the Scotch Whisky Research Institutue. Before experiment commencement and after at least every 3rd distillation, the stainless stripping still was cleaned by distilling 2% (80 mL of 50% caustic topped off to 2 L) caustic solution (50286, Chemstation) for 30 min, then scrubbed with an abrasive pad, and finally washed thoroughly with RO water. Before commencement and after at least every 3^rd^ distillation, the copper spirit still was cleaned by distilling 2% (40 mL of 50% caustic topped off to 1 L) caustic solution (50286, Chemstation) for 15 min. The heat was then turned off and the caustic was soaked for an additional 15 min, after which the still pot and swan neck were scrubbed with an abrasive pad and washed thoroughly with RO water.

This experimental design resulted in ten treatments (3 corn varieties x 3 terroirs + 1 corn variety/1 terroir [REV25BHR26 from Hansford County, TX]), and each treatment was processed in biological triplicates, creating 30 batches total.

### HPLC analysis of mash and beer

HPLC was used to detect compounds DP4+ (dextrins), DP3 (maltotriose), maltose, glucose, lactic acid, glycerol, acetic acid, and ethanol in mash and beer samples at various time points. Each of the 30 batchs were analyzed at various timepoints with HPLC, with each timepoint being analyzed in triplicates. The HPLC triplicates were assessed to ensure the relative standard deviation was below 0.5%, and then averaged to achieve a final value for statistical analysis. Standards were run before every monitored timepoint. The standard for the HPLC was Ethanol Industry HPLC Standard (Midland Scientific Inc., La Vista, NE, USA), and includes the following compounds: DP4+ (dextrins), DP3 (maltotriose), maltose, glucose, lactic acid, glycerol, acetic acid, and ethanol.

All HPLC analyses in this study were executed as described previously [37]. Briefly, samples were centrifuged at 4000 x *g* using a desktop centrifuge, and then filtered through a 0.22-μm membrane filter. An autosampler vial containing at least 0.5 mL of the sample was analyzed by HPLC using a Shimadzu LT-20AT (Shimadzu USA, Canby, OR). The separations were carried out using a Rezex ROA-Organic Acid H+ 8% (300×7.8 mm, 5 μm, Phenomenex, Torrance, CA, USA). The HPLC analysis was performed in isocratic mode with a mobile phase of 0.005 N sulfuric acid using vacuum sealed, pre-made solvent (Chata Biosystems). The analytes were detected by refractive index (RID-20A, Shimadzu USA, Canby, OR).

### New-make bourbon and corn kernel descriptive sensory analysis

A human sensory panel was used in this research. All participants signed a written consent form after being walked through their rights as participants, and the Texas A&M institutional review board specifically approved the study (IRB Number: IRB2016-0842M).

A whiskey lexicon was developed based on 28 commodity spirits (14 whiskeys from different grain origins, 15 miscellaneous spirits) and 21 new-make spirits. The focus was on whiskey and new-make whiskey, but other miscellaneous spirts (cachaça, vodka, rum, ouzo, vermouth, gin, Sambuca, flavored liqueurs, triple sec, and amaretto) were used to cover attributes not commonly found in whiskey or new-make spirits. Other sources used to develop attributes were from new-make spirit published literature [38,39,40,41,42,43, 44,45,46,47,48,49,50,51,52,53] and existing, published lexicons [54,55] to encompass alcohol and spirits, but the developed lexicon focused on flavors and aromas found in new-make bourbon. New-make bourbon and corn were evaluated by a 7-member, expert trained whiskey aroma descriptive attribute panel that has over 20 years of experience in descriptive sensory attribute evaluation across food products. Aroma analysis allows for a nearly full assessment of a whiskey’s flavor, negating any effects of alcohol ingestion, and therefore is the main form of sensory evaluation used in the industry [56]. This panel helped develop and was trained using the new-make bourbon lexicon for 31 days, on various attributes as described in Table 2, followed by a validation trial prior to testing. Following the completion of the new-make bourbon samples, panelists trained for 3 days on corn samples using the new-make bourbon lexicon. Whiskey and corn aroma attributes were measured using a new-make bourbon lexicon (0 = none and 15 = extremely intense) that was specifically developed for this research. After training was complete, panelists were presented three to four new-make samples per day for 8 days, and six corn samples a day for 5 days in a two-hour session. Panelists evaluated new-make samples individually, and reached consensus on attributes and intensities. Prior to the start of each trained panel corn evaluation day, panelists were calibrated using one orientation or “warm up” sample that was evaluated and discussed orally. After evaluation of the orientation sample, panelists were served the first sample of the session and asked to individually rate the sample for each corn/new-make bourbon aroma lexicon attribute. References were available at all times during training and evaluation. Steamed cotton towels were available for cleansing the nasal palette during evaluation of samples. New-make samples were prepared no more than 30 minutes prior to serving by diluting the new-make bourbon (~125 proof, 62.5% alcohol by volume) with double-distilled water to testing strength used in the industry (40 proof, 20% alcohol by volume [56]). Each panelist was served 8 mL of the diluted sample in a nosing glass (grappa or tulip glass), with a watch glass to concentrate volatiles. Corn samples were ground one hour prior to serving. Each panelist was served 10 g of milled corn sample in a medium snifter glass covered with a watch glass to concentrate volatiles. Samples were identified with random three-digit codes and served in random order.

**Table 2:** New-make bourbon lexicon.

Aroma	Description	Reference Scale	Reference Preparation
AROMA FACTORS			
Alcohol	A colorless, pungent, chemical-like aromatic associated with distilled spirits or grain products.	5.0: Absolut Vodka (40% ABV)	Dilute 16 mL of Absolut Vodka in 64 mL of distilled water. Serve 15 mL in a snifter. Cover.
		8.0: Barsol Pisco (41.3% ABV)	Serve 15 mL of Barsol Pisco Spirit in a snifter. Cover.
		10.0: Grain Neutral Spirit (60%)	Dilute 100 g of 190 proof neutral spirit in 77.25 g of distilled water. Serve 15 mL in snifter. Cover.
		12.0: Grain Neutral Spirit (90% ABV)	Serve 15 mL of 190 proof neutral spirit in snifter. Cover.
Anise	A colorless, pungent, chemical-like aromatic associated with distilled spirits or grain products.	7.5: Anise Seed	Place ½ teaspoon of McCormick’s anise seed in a snifter. Cover.
Banana	Aromatic characteristic of ripe bananas.	10.0: Banana Extract	Place 1 drop of banana extract on a cotton ball. Serve in snifter glass. Cover.
Barnyard	Aromatic characteristic of livestock animal housing.	6.0: McCormick’s Ground White Pepper	Place ½ teaspoon of white pepper in 1 ounce of distilled water.
Blended	The melding of individual sensory notes such that the products present a unified overall sensory experience as opposed to spikes or individual notes.	3.0: Absolut Vodka (40% ABV)	Dilute 16 mL of Absolut Vodka in 64 mL of distilled water. Serve 15 mL in a snifter. Cover.
		5.0: McCormick Gin (40% ABV)	Serve 15 mL of McCormick Gin in a snifter. Cover.
		10.0 Tanqueray Gin (47.3% ABV)	Serve 15 mL Tanqueray Gin in a snifter. Cover.
Brown Spice Complex	The sweet, brown aromatic associated with spices such as cinnamon, clove, nutmeg, and allspice.	3.0: Cinnamon Stick	Place 1 cinnamon stick (1/2 teaspoon) in a 2-ounce glass jar with screw-on type lid.
		7.0: Whole Nutmeg & Clove Bud	Place 1 whole nutmeg (2 teaspoons) and 3 clove buds (1/4 teaspoon) in a 2-ounce glass jar with screw-on type lid.
Brown Sugar	A rich, full, round, sweet aromatic impression characterized by some degree of darkness.	6.0: C&H Pure Cane Sugar, Golden Brown	Place 1 teaspoon brown sugar in a snifter. Cover.
Burnt	The dark brown impression of an over-cooked or over-roasted product that can be sharp, bitter, and sour.	4.5: Benzyl Disulfide	Place 0.1 gram of benzyl disulfide in a covered soufflé cup.
		8.0: Puffed Wheat Cereal	Serve 1 tablespoon of cereal in a covered soufflé cup.
Buttery	Aromatic associated with fresh butter fat, sweet cream.	5.0: McCormick Extract	Place 1 drop of coconut extract on a cotton ball. Serve in snifter glass. Cover.
		7.0: Land O’Lakes Unsalted Butter	Place ½ tablespoon in a covered snifter.
Butyric	An aroma associated with butyric acid, cheesy, also sickly.	6.0: Butyric Acid	Place 1 drop of butyric acid to a cotton ball. Serve in a snifter glass. Cover.
Caramel	A round, full-bodied, medium brown, sweet aromatic associated with cooked sugars and other carbohydrates. Does not include burnt or scorched notes.	8.0: Le Nez du Café no.25 “caramel”	Place 1 drop of essence on a cotton ball in a soufflé cup. Cover.
Cardboard/Paper-like	The aromatic associated with cardboard or paper packaging.	3.0: White Napkin	Place a 2-inch napkin piece in a soufflé cup.
		7.5: Cardboard	Cut a 2-inch square of cardboard. Place in a covered soufflé cup.
Coconut	The slightly sweet, nutty, somewhat woody aromatic associated with coconut.	7.5: McCormick Extract	Place 1 drop of coconut extract on a cotton ball. Serve in snifter glass. Cover.
Coffee	An aroma note associated with coffee.	3.0: Werther’s Coffee	Place a single, unwrapped Werther’s Coffee Flavored Caramel in a snifter. Cover.
		8.0: Folgers Instant Coffee Crystals	Place 1/8 of a teaspoon of Folgers Instant Coffee Crystals.
Corn	An aroma note associated with corn.	5.0: Canned corn	Drain and rinse canned corn and serve in soufflé cup.
		8.0: Amoretti Sweet Corn Essence	Place 1 drop of Amoretti Sweet Corn Essence on cotton ball and place in soufflé cup.
Fermented/Yeasty	The pungent, sweet, slightly sour, sometimes yeasty, alcohol-like aromatic characteristic of fermented fruits or sugar or over-proofed dough.	5.0: Guinness Extra Stout Beer	Serve 15 mL Guinness Extra Stout Beer in a covered glass.
Fruity-Berry	The sweet, sour, floral, sometimes heavy aromatic associated with a variety of berries such as blackberries, raspberries, blueberries, or strawberries.	3.0: Captain Morgan Rum	Serve 15 mL in a covered glass.
		6.0: Tropicana Berry Juice	Serve 15 mL in a covered glass.
		10.0: Private Selection Triple Berry Preserves	Place 1 teaspoon of jelly in a medium snifter. Cover.
Fruity-Citrus	A citric, sour, astringent, slightly sweet, peely, and somewhat floral aromatic that may include lemons, limes, grapefruits, or oranges.	4.5: Lemon peel + lime peel	Put 0.5 grams lemon peel and 0.5 grams lime peel in a medium snifter. Cover.
		7.5: Grapefruit peel	Put 0.25 grams grapefruit peel in a medium snifter. Cover.
Fruity-Dark	An aromatic impression of dark fruit that is sweet and slightly brown and is associated with dried plums and raisins.	3.0: Sunsweet Amaz!n Prune Juice	Mix 1 part juice with 2 parts water. This may be prepared 24 hours in advance and refrigerated. Bring to room temperature.
		4.5: Sun-Maid Prunes	Chop 1/2 cup prunes. Add ¾ cup of water and cook in microwave on high for 2 minutes. Filter with a sieve. Place 1 tablespoon of juice in a medium snifter. Cover.
		6.0: Sun-Maid Raisins	Chop 1/2 cup of raisins. Add ¾ cup water and cook in microwave on high for 2 minutes. Filter with a sieve. Place 1 tablespoon of liquid juice in a medium snifter. Cover.
Fruity-Other	A sweet, light, fruity, somewhat floral, sour, or green aromatic that may include apples, grapes, peaches, pears, or cherries.	5.0: Le Nez du Café n. 17 “apple”	Place 1 drop on a cotton ball in large snifter. Cover.
		9.0: Effen Black Cherry Vodka	Serve 15 mL in a covered glass.
Fishy	Aromatic associated with trimethylamine and old fish.	7.0: Canned tuna	Place 1 gram of tuna from can in a covered soufflé cup.
Floral	A sweet, light, slightly fragrant aromatic associated with flowers.	6.0: Welch’s 100% White Grape Juice	Mix 1 part water and 1 part juice. Place 15 mL of mixture in a snifter. Cover.
		8.0: Le Nez du Café n.12 “coffee blossom”	Place 1 drop of Le Nez du Café essence on a cotton ball in a snifter. Cover.
Grain Complex	The light brown, dusty, musty, sweet aromatic associated with grains.	5.0: Rice & Wheat	Blend ½ cup of Rice Chex and ½ cup of Post Shredded Wheat in a food processor. Serve 1 tablespoon in a snifter. Cover.
		8.0: Georgia Moon Corn Whiskey	Serve 15 mL in a snifter. Cover.
Green	An aromatic characteristic of fresh, plant-based material. Attributes may include leafy, viney, unripe, grassy, and peapod.	9.0: Parsley water	Rinse and chop 25 grams of fresh parsley. Add 300 milliliters of water. Let sit for 15 minutes. Filter out the parsley. Serve 1 tablespoon of the water in a snifter. Cover.
Hay-like	The lightly sweet, dry, dusty aromatic with slight green character associated with dry grasses.	7.5: McCormick Parsley Flakes	Place 1 teaspoon of flakes in a medium snifter. Cover.
Herb-like	The aromatic commonly associated with green herbs that may be characterized as sweet, slightly pungent, and slightly bitter. May or may not include green or brown notes.	3.0: McCormick Bay Leaves, ground thyme, basil leaves	Mix together 0.5 grams of each herb. Break the bay leaves into smaller pieces with your hands first, and then grind all the herbs together using a mortar and pestle. Add 100 milliliters of water. Mix well. Put 5 milliliters of herb water in a medium snifter, and add 200 milliliters of water. Serve 1 oz. in soufflé cup.
		10.0: McCormick Bay Leaves, ground thyme, basil leaves	Mix together 0.5 grams of each herb. Break the bay leaves into smaller pieces with your hands first, and then grind all the herbs together using a mortar and pestle. Add 100 milliliters of water. Mix well. Serve 1 oz. in soufflé cup.
Honey	Sweet, light brown, slightly spicy aromatic associated with honey.	6.0: Busy Bee Pure Clover Honey	Dissolve 1 tablespoon of honey in 250 mL of distilled water. Serve 15 mL in snifter. Cover.
Lactic Acid	A sour aroma note associated with lactic acid.	5.0: Buttermilk	Serve 1 oz. buttermilk in soufflé cup.
		8.0: Sauerkraut	Serve 5 g sauerkraut in soufflé cup.
Leather	An aromatic associated with tanned animal hides.	3.0: Leather Shoe Lace	Place a 3-inch length of leather shoe lace in a covered snifter.
		10.0: Hazels Gifts Leather Essence	Place 2 drops on a cotton ball in a covered snifter.
Malt	The light brown, dusty, musty, sweet, sour and or slightly fermented aromatic associated with grains.	3.5: Post Grape Nut Cereal	Serve Post Grape-Nut Cereal in a covered snifter.
		6.0: Carnations Malted Milk	Place ½ teaspoon in a covered snifter.
Medicinal	A clean, sterile aromatic characteristic of antiseptic-like products such as Band-Aids, alcohol, and iodine.	6.0: Le Nez du Café no. 35 “medicinal”	Place 1 drop of essence on a cotton ball in a soufflé cup.
		8.0: Tanqueray Gin	Serve 15 mL of Tanqueray Gin in covered glass.
		12.0: Iodine	Serve 1:1 iodine and distilled water solution in a covered glass (50 mL iodine tincture, 50 mL distilled water.
Mint	An aromatic with mint family (sweet, green, and menthol).	4.0: Absolut Vodka/Mint Gum	Place 3 stick of mint gum in 150 mL of Absolut Vodka and let steep for 30 minutes. Serve 15 mL Absolut Vodka in covered glass.
		8.0: Listerine	Serve in a covered snifter.
Molasses	An aromatic associated with molasses; has a sharp, slight sulphur and/or caramelized character.	6.5: Black Strap Molasses	Mix 2 teaspoons of molasses in 250 milliliters of water. Serve ¼ cup in a mason jar. Cover.
Musty/Dusty	The aromatic associated with dry, closed-air spaces such as attics and closets. May have elements of dry, musty, papery, dry soil, or grain.	5.0: Kretschmer Wheat Germ	Serve 1 tablespoon wheat germ in a medium snifter. Cover.
		10.0: 2,3,4-Trimethoxybenzaldehyde	Place 0.1 gram in a medium snifter. Cover.
Musty/Earthy	The somewhat sweet, heavy aromatic associated with decaying vegetation and damp, black soil.	3.0: Mushrooms	Place 2, washed 1/2 –inch cubes in a covered snifter.
		9.0: Miracle Gro Potting Soil	Fill a 2-ounce glass jar half full with potting soil and seal tightly with screw-on type lid.
		12.0: Le Nez du Café no. 1 “earthy”	Place 1 drop of essence on a cotton ball in a large snifter. Cover.
Nutty	A slightly sweet, brown, woody, oily, musty, astringent, and bitter aromatic commonly associated with nuts, seeds, beans, and grains.	7.5: Le Nez du Café no. 29 “roasted hazelnut”	Place 1 drop of essence on a cotton ball in a covered glass.
		9.0: Almont/Walnut Puree	Puree the almonds and walnuts separately in blenders for 45 seconds on high speed. Combine equal amounts of the chopped nuts. Serve in a covered glass.
Oily	An overall flavor term for the aroma and flavor notes reminiscent of vegetable oil or mineral oil products.	9.0: Vegetable Oil	Serve vegetable oil in a covered glass.
Overall Sweet/Sweet Aromatics	The perception of a combination of sweet substances and aromatics.	3.0: Vanillin	Mix 0.5 g of vanillin into 250 mL of water in covered snifter.
		5.0: Vanillin	Mix 2 g of vanillin into 250 mL of water in covered snifter.
Overall Sour/Sour Aromatics	An aromatic associated with the impression of a sour product.	2.0: Bush’s Pinto Beans, canned	Drain and rinse with distilled water, 1 tbsp. placed in covered snifter.
		5.0: Buttermilk	Serve 1 oz. buttermilk in a covered glass.
Pepper	The spicy, pungent, musty, and woody aromatic characteristic of ground black pepper.	13.0: McCormick Ground Black Pepper	Place ½ teaspoon pepper in a medium snifter. Cover.
Rancid	Aromatic associated with oxidized fats and oils.	5.0: Vegetable oil (oxidized/rancid)	Keep oil in an open container or a warm storage place for 1 week. Place 1 oz. rancid oil in covered glass.
Roast	Dark brown impression characteristic of products cooked to a high temperature by dry heat. Does not include bitter or burnt notes.	6.0: Le Nez du Café no. 34 “Roasted Coffee”	Place one drop on cotton ball. Place in covered glass.
Smokey	An acute, pungent aromatic that is a product of the combustion of wood, leaves, or a non-natural product.	6.0: Diamond Smoked Almonds	Place 5 almonds in a covered snifter.
Soapy	An aroma associated with unscented soap.	6.5: Ivory Soap Flakes	Place 0.5 g bar soap in 100 ml of room temperature water. Serve in large snifter, covered snifter.
Solvent-like	General term used to describe many classes of solvents, such as acetone, turpentine, chemical solvents, etc.	5.0: Acetone solution	Dilute 10 mL acetone in 100 mL distilled water until dissolved, and serve in 2 oz. soufflé cup. Cover.
		8.0: Lighter fluid solution	Dilute 10 mL of lighter fluid in 100 mL distilled water until dissolved, and serve in 2 oz. soufflé cup. Cover.
Stale	The aromatic characterized by a lack of freshness.	4.5: Mama Mary’s Gourmet Original Pizza Crust	Serve cut a 2-inch square of crust and serve in soufflé cup. Cover.
Sulphur	Aromatic associated with hydrogen sulfide, rotten egg.	3:0: Bush’s Pinto Beans	Drain and rinse the beans. Serve 1 tbsp. in a covered glass.
		11.0: Dimethyl Trisulfide	Dilute 1 ml of dimethyl trisulfide in 100 ml distilled water until dissolved, and serve in 2 oz. soufflé cup. Cover.
		15.0: Dimethyl Trisulfide	Place 1 drop of dimethyl trisulfide on a cotton ball. Serve in a soufflé cup. Cover.
Tobacco	The brown, slightly sweet, slightly pungent, fruity, floral, spicy aromatic associated with cured tobacco.	5.0: Le Nez du Café no. 33 “pipe tobacco”	Place 1 drop of essence on a cotton ball in a large snifter. Cover.
		7.0: Marlboro Cigarettes, southern cut	Break cigarette and place 0.1 grams tobacco in a medium snifter. Cover.
Vanilla	A woody, slightly chemical aromatic associated with vanilla bean, which may include brown, beany, floral, and spicy notes.	2.5: Le Nez du Café no.10 “vanilla”	Place 1 drop of Le Nez du Café essence on a cotton ball in a snifter glass. Cover.
		5.5: Spice Islands Bourbon Vanilla Bean	Place 0.5 gram chopped vanilla beans in a snifter glass. Cover.
Vinegar	A sour, astringent, slightly pungent aromatic associated with vinegar or acetic acid.	2.0: 0.5% acetic acid solution	Dilute 5 mL distilled white vinegar in 1000 mL distilled water. Serve in soufflé cup. Cover.
		3.0: 2.0% acetic acid solution	Dilute 20 mL of white distilled vinegar in 1000 mL distilled water. Serve in soufflé cup. Cover.
Woody	The sweet, brown, musty, dark aromatic associated with a bark of a tree.	4.0: Diamond Shelled Walnuts	Serve 1 tablespoon of chopped walnuts in a snifter. Cover.
		7.5: Popsicle Sticks	Break popsicle sticks in two and place in snifter. Cover.
NASAL FEELING FACTORS			
Nose Cooling	The chemical feeling factor or sensation of cooling in the nasal passages when sniffing.	6.0: Tanqueray Gin	Serve 15 mL in covered glass.
		8.0: Listerine solution	Mix 1:1 dilution Listerine and distilled water; serve in soufflé cups.
		12.0: Listerine	Serve 1 oz. in a covered glass.
Nose Drying	The chemical feeling factor or sensation of drying in the nasal passages when sniffing.	4.0: Barrelstone Cellars Merlot, 2013	Serve 15 mL Barrelstone Cellars Merlot 2013 in covered glass
		6.0: Grain Neutral Spirit (60% ABV)	Add 100 g of Grain Neutral Spirit to 77.25 g distilled water; serve in covered glass.
		8.0: Unscented Hand Sanitizer	Serve 1 oz. in a covered glass.
Nose Warming	Chemical feeling factor described as a warmth or burning sensation in the nasal passages occurring when sniffing.	3.0: Barrelstone Cellars Merlot, 2013	Serve 15 mL Barrelstone Cellars Merlot 2013 in covered glass.
		7.0: TX Blended Whiskey (41% ABV)	Serve 15 mL F&R TX Whiskey Blend in covered glass.
		9.0: Grain Neutral Spirit (60% ABV)	Add 50 g of Grain Neutral Spirit to 79.8 g distilled water; serve in covered glass.
		12.0: Grain Neutral Spirit (60% ABV)	Add 100 g of Grain Neutral Spirit to 77.25 g distilled water; serve in covered glass.
Prickle/Pungent	A feeling factor that can range from tingling or irritating, sharp, physically penetrating sensation of the nasal cavity.	5.0: Horse Radish Solution	Serve 1/8 teaspoon in a covered glass.
		7.0: Captain Morgan Rum	Serve 15 mL Captain Morgan in a covered snifter.
		9.0: McCormick Ground Black Pepper	Serve ½ teaspoon cracked pepper in a covered glass.
		10.0: Horse Radish Sauce solution	Mix 5 g horseradish sauce in 30 mL distilled water; serve 1 oz. in labeled soufflé cups.

### New-make bourbon and corn congener identification and quantification

Volatiles were captured from the same new-make bourbon and corn samples evaluated by the expert, trained descriptive panel. After samples were prepared for panelists, approximately 80 g of new-make bourbon and 40 g of corn were placed in glass jars (473 mL, new-make; 236 mL, corn) with a Teflon lid under the metal screw-top to avoid off-aromas. The headspace was collected with a solid-phase micro-extraction (SPME) portable field sampler (Supelco 504831, 75 μm Carboxen/ polydimethylsiloxane, Sigma-Aldrich, St. Louis, MO, USA). The headspace above each new-make and corn sample in the glass jar was collected for 2 hours for each sample at room temperature at approximately 21°C; new-make samples were mixed at low speeds on a laboratory stirrer hot plate (Model P.C.- 351,120 V, Corning Glass Works, Corning, NY, USA).

Volatiles were evaluated using a gas chromatograph/mass spectrometer system with dual sniff ports for characterization of aromas (GC-MS/O). This technology provided the opportunity to separate individual volatile compounds, identify their chemical structure and characterize the aroma/flavor associated with the compound. Upon completion of collection, the SPME was injected in the injection port of an Agilent Technologies (Santa Clara, CA, USA) 7920 series GC where the sample was desorbed at 280°C. The sample was then loaded onto the multi-dimensional gas chromatograph into the first column (30m X 0.53mm ID/ BPX5 [5% Phenyl Polysilphenylene-siloxane] X 0.5 μm, SGE Analytical Sciences, Austin, TX, USA). The temperature started at 40°C and increased at a rate of 7°C/minute until reaching 260°C. Upon passing through the first column, compounds were sent to the second column ([30m X 0.53mm ID; BP20- Polyethylene Glycol] X 0.50 μm, SGE Analytical Sciences, Austin, TX, USA). The gas chromatography column then split into three different columns at a three-way valve with one going to the mass spectrometer (Agilient Technologies 5975 Series MSD, Santa Clara, CA) and two going to the two humidified sniff ports with glass nose pieces heated to 115°C. The sniff ports and software for determining flavor and aroma were part of the AromaTrax program (MicroAnalytics-Aromatrax, Round Rock, TX, USA). The GC-MS/O set-up could host two operators, and to keep a human variable constant, the same two operators always evaluated the volatiles. These two flavor chemistry research technicians underwent sensory training using the lexicon developed here; were trained on aroma identification, quantification, and GC-MS/O operation; and had previously analyzed over 500 hours of GC-MS/O samples. Each operator was trained to accurately use the Aromatrax software to indicate where an aroma event was present. Only those volatile compounds that were present during an aroma event (where any detectable aroma was present at the sniff port) were kept for analysis. Aroma identity was not collected for each of the volatile compounds.

The MS detected ions within 35–300 m/z range in the electron impact mode at 70 eV. Chromatography data was collected in the scan mode (Agilent MSD Chemstation E.02.02.1431 software, Agilent Technologies, Santa Clara, CA, USA). Volatile compounds with at least 1200 total ion counts (area under the curve) and a quality score above 75 (based on its match to the NIST library) and were present during an aroma event were kept for analysis. Units of measure were total ion count (TIC) area under the curve and compound identity was based on the NIST library. For verification of volatile compound identification (via retention times) and quantification, alkane standards (C7 to C30; Catalog #49451-U; Sigma Aldrich, St. Louis, MO, 63103) were run prior to and after experimental samples to verify the retention times and concentrations were consistent among samples.

### Proximate analysis of corn kernels

Proximate analysis of corn samples were determined from each variety x terroir treatment. Fourier Transform Near-Infrared Reflectance (FT-NIR) Spectroscopy was used for predicted values of protein, starch, and lipid of the corn samples. Whole kernels and ground corn samples were evaluated with a Thermo Scientific Antaris II FT-NIR (Thermo Fischer Scientific) using a sample spinner cup that held approximately 175g of whole kernel corn. Preparation of ground samples was as described previously [57]. Approximately 175 grams of each corn sample were ground to 2 mm using a Polymix PX-MFC 90 D mill (Kinematica Ag, Eschbach, Germany) and further ground using a Cyclone sample mill (UDY Corporation, Fort Collins, CO, USA) to 1-mm fineness. The first set of 10 whole corn samples were run in triplicate with 128 scans and 10 ground corn samples were run in triplicate with 64 scans at ambient temperature. Reflectance measurements were taken by using a rotating cup that holds approximately 175g of corn over the instrument’s integrating sphere module. Approximately, 3000 points across the spectrum, every 4 wave numbers, were collected for each sample scanned at a spectral range between 10,000 to 4,000 cm^-1^. The predictions were made with calibrations created using primarily Texas grown corn and wet chemistry performed by Ward Laboratories (Kearny, NE, USA). Whole and ground kernel calibrations were developed using the same samples, ground kernel calibrations are better but are also destructive to the grain.

### Statistical analyses

The goal was to attribute variability to variety, terroir, the interaction of these effects, as we were attempting to draw conclusions for possible levels of Texas terroirs and possible levels of commercial yellow dent hybrids. Our interest was not solely concerned with the levels of Hansford County, Hidalgo County, Hill County, and Calhoun County for terroir; or the levels of D57VP51—Dyna-Gro, 2C797—Mycogen Seed, and REV25BHR26—Terral Seed for variety. Therefore, the data was analyzed as a completely randomized design, using variety, terroir, and their interaction as random effects for all Restricted Maximum Likelihood (REML) models using JMP12 (SAS Institute, Inc. Cary, NC, USA). Variance components and percent of total variance were obtained from these REML analyses, which were used to explain the impact of variety and terroir on variation. Correlation probabilities, analysis of variance (ANOVA), and regression analyses were obtained using JMP12 (SAS Institute, Inc. Cary, NC, USA).

## Results and discussion

### Corn analysis

#### Protein, fat, and starch

FT-NIR was used to measure the percentage of protein, fat, and starch (dry basis) in both whole and ground corn kernels. Whole kernel analysis is the most common method used by distillers when analyzing kernels after harvest, before loading into a silo for storage, and upon delivery to the distillery.

Whole corn analysis showed that nearly 85% of the experimental variation in protein among the treatments was due to variety, terroir, and interaction effects (Table 3); the rest of the variation was residual, also known as unexplained error variance, and here reported as replicates nested within variety and terroir. However, terroir was responsible for 0% of the variation in, and instead variety and interaction effects accounted for over 75% of fat and starch variation.

**Table 3:** Percent of total variance for proximate analysis of corn kernels as determined through REML.

Effect	Whole Corn			Milled Corn		
	Protein %	Fat (Oil) %	Starch %	Protein %	Fat (Oil) %	Starch %
Terroir	39.1%	0%	0%	60.8%	49.9%	2.7%
Variety	26.5%	61.6%	28%	17.4%	32.8%	9.4%
Variety*Terroir	19.1%	16.6%	48.4%	21.5%	15.5%	85.6%
Replicate[Variety,Terroir]	15.3%	21.8%	23.6%	0.3%	1.8%	2.3%
Sum Total Variance	100%	100%	100%	100%	100%	100%
Units	Mg g^-1^	Mg g^-1^	Mg g^-1^	Mg g^-1^	Mg g^-1^	Mg g^-1^
Mean Value +/- SE	7.86 +/- 0.46	4.30 +/- 0.14	68.02 +/- 0.41	9.35 +/- 0.38	3.21 +/- 0.19	69.01 +/- 0.20
Observations (n)	30	30	30	30	30	30

Distillers grind kernels in a mill to create a grist prior to mashing. Nearly 100% of the experimental variation in milled corn protein was due to variety, terroir, and interaction effects, with residual variation having essentially no role (Table 3). The reduced residual is almost certainly due to greater precision of the milled corn. Unlike with whole corn analysis, terroir was responsible for variation in fat, with variety and interaction effects having a lesser but still substantial role. Variation in starch was largely due to interaction effects, with terroir, variety, and residual effects playing a small role in variation.

These results for milled corn analysis were not well aligned with whole corn analysis. It is well known that grinding helps to homogenize samples, improving results in near infrared reflectance spectroscopy, and that these particular FT-NIRS calibrations and predictions work better in ground samples than whole samples [57]. While discrepancies in variance components between whole corn and milled corn exist, the proximate analysis results indicate that variety, terroir, and interaction effects are responsible for most of the variation in protein, fat, and starch levels. Given that starch ultimately dictates the amount of alcohol that can be produced, and that protein and fat are potentially important for flavor (e.g., amino acids are important for fusel alcohol production via the Ehrlich pathway [58]), these results suggest that alcohol yield and flavor could be impacted by variety and terroir in our samples.

#### Congeners and aromas in milled corn kernels

Milled corn samples were exposed to GC-MS/O and descriptive sensory analysis techniques. The GC-MS/O detected 52 different congeners that registered an aroma event via olfactometric detection by a trained operator. Descriptive sensory analysis utilized a trained sensory panel to detect and quantify up to 49 different corn kernel aromas. The percent of total variance is reported for congeners (Table 4) and aromas (Table 5) where the residual effect was responsible for no more than ~80% of the variation. For the 44 compounds and 37 aromas detected but not shown (S1 Table), random residual error was responsible for most of the variation suggesting a low importance of terroir or variety.

**Table 4:** Percent of total variance for milled corn kernel congener concentrations as determined through REML.

Effect	GC-MS/O							
	Acetic Acid	Benzaldehyde	Hexanal	Methane, thio-bis	1-Hexanol	Ethyl decanoate	3-Dodecen-1-al	Decanal
Terroir	0%	11.9%	0%	4%	25.1%	4%	5.5%	0%
Variety	19.1%	19.9%	0%	0%	0%	17%	6.4%	8%
Variety*Terroir	0%	37.5%	24.4%	30%	1.6%	1%	10.1%	49.3%
Replicate[Variety,Terroir]	80.9%	30.7%	75.6%	66%	73.3%	78%	78%	42.7%
Sum Total Variance	100%	100%	100%	100%	100%	100%	100%	100%
Units	Ion Count	Ion Count	Ion Count	Ion Count	Ion Count	Ion Count	Ion Count	Ion Count
Mean Value +/- SE	65197.7 +/- 19981.3	6877.6 +/- 2900.2	280095.9 +/- 78261.2	60219.4 +/- 38995.7	18681.67 +/- 12727.3	62152.3 +/- 47293.6	27218.5 +/- 14948.6	4989.4 +/- 3947.3
Observastions (n)	30	30	30	30	30	30	30	30

Ion Count **=** Total ion count (TIC) area under the curve and compound identity was based on the NIST library.

**Table 5:** Percent of total variance for milled corn kernel aroma concentrations as determined through REML.

Effect	Descriptive Sensory Analysis											
	Overall Sweet	Overall Sour	Corn	Woody	Oily	Rancid	Medicinal	Leather	Barnyard	Soapy	Solvent Like	Butyric
Terroir	1.8%	10.8%	20%	31.9%	31.2%	34.2%	0%	0%	53.2%	37.1%	23.6%	21.3%
Variety	24.6%	0%	0%	0%	0%	4.6%	0%	22.1%	0%	0%	0%	0%
Variety*Terroir	0%	20.3%	27.4%	0%	13.4%	0%	30.5%	8.9%	0%	0%	0%	0%
Replicate[Variety,Terroir]	73.6%	68.9%	51.6%	68.1%	55.4%	61.2%	69.5%	69%	46.8%	62.9%	76.4%	78.7%
Sum Total Variance	100%	100%	100%	100%	100%	100%	100%	100%	100%	100%	100%	100%
Units	Intensity	Intensity	Intensity	Intensity	Intensity	Intensity	Intensity	Intensity	Intensity	Intensity	Intensity	Intensity
Mean Value +/- SE	2.8 +/- 0.2	2.5 +/- 0.3	6.9 +/- 0.3	6.4 +/- 0.3	2.3 +/- 0.2	0.4 +/- 0.2	1.8 +/- 0.1	1.6 +/- 0.2	1.0 +/- 0.4	0.3 +/- 0.2	0.2 +/- 0.1	0.1 +/-0.1
Observations (n)	30	30	30	30	30	30	30	30	30	30	30	30

Intensity corresponds to an aroma intensity, with a scale of 0–15, and was determined by the trained sensory panel.

Two congeners where the variation was not largely due to a residual effect appeared especially important throughout the study—benzaldehyde and decanal. Benzaldehyde possesses a characteristic almond-like aroma [59], and decanal imparts fruity aromas [60]. Interaction effects were responsible for most of the variation found in these two aldehydes.

For the aromas detected via descriptive sensory analysis and highlighted in Table 5, residual effects on average accounted for the majority of the variation (a range of about 50–75%). That said, of the twelve aromas highlighted, the non-residual experimental variation was largely due to terroir in seven (Woody, Oily, Rancid, Barnyard, Soapy, Solvent Like, Butyric), variety in two (Overall Sweet, Leather), and the interaction in three (Overall Sour, Corn, Medicinal).

Overall, kernel analysis thus indicated that we might expect variety and terroir to influence alcohol yield and flavor. Next, we aimed to process each kernel treatment into mash, beer, and finally new-make bourbon, conducting relevant analyses at each step.

### Mash and fermentation analysis

#### Specific gravity

**Fig 1:**
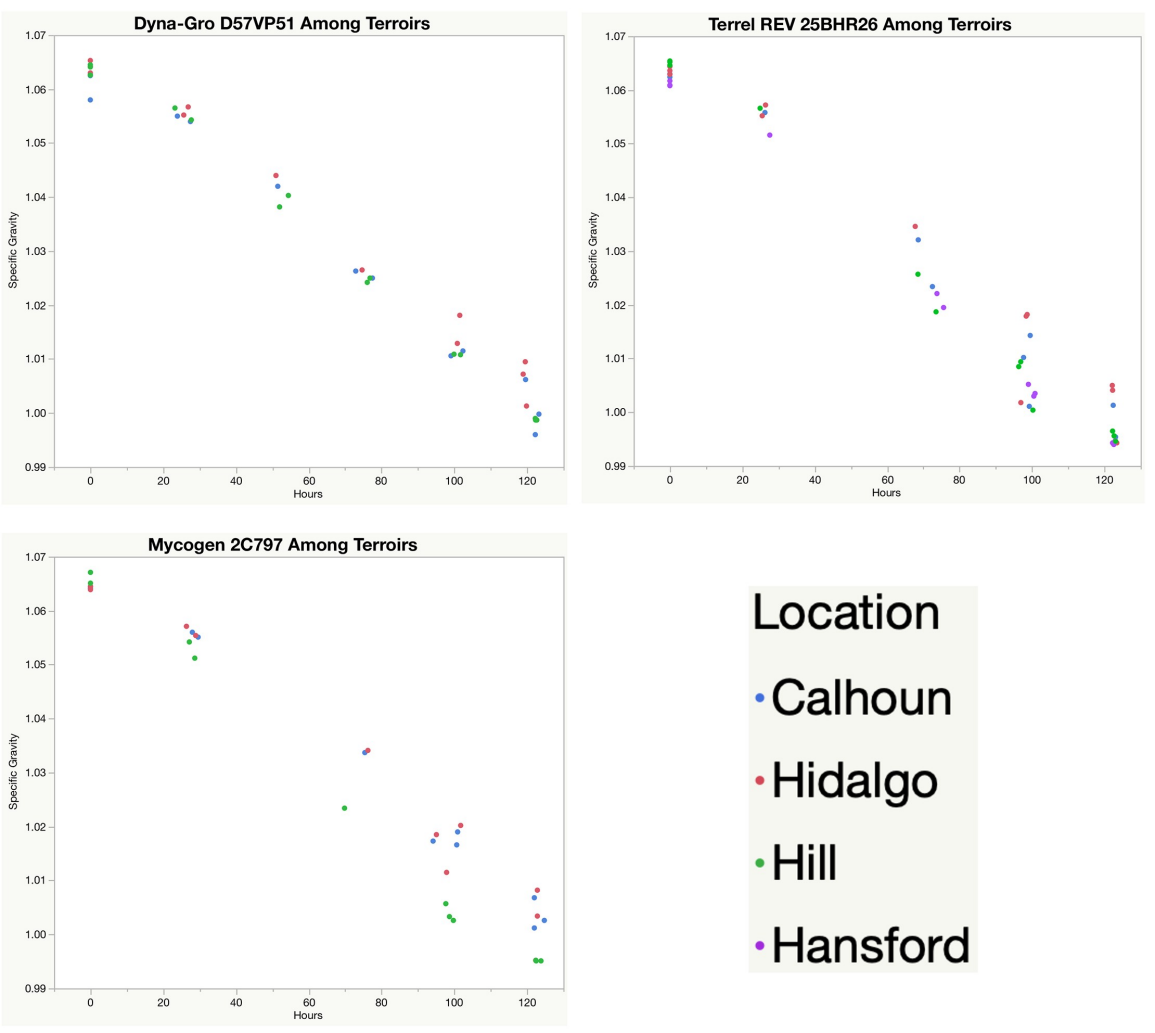
Fermentation growth curves using specific gravity as the response factor. Higher levels of specific gravity at the end of fermentation indicate lower attenuation, which will potentially lead to a lower alcohol yield.

Analysis of covariance (ANCOVA) was carried out with treatment, hours into fermentation, and the interaction as effects, and specific gravity as the response. A visual of fermentation growth curves, as measured as specific gravity, for each variety among the different terroirs (Fig 1, Table 6) shows that the different treatments displayed significantly different slopes (p = 0.0046). Table 7 shows the percent of total variance for specific gravity at different points during mashing and fermentation. At all timepoints, the terroir was the biggest driver of variation next to the residual variation. The variety in this case contributed meaningful but less overall variation than the terroir or replicate variation. This is likely due to the samples being from relatively narrow germplasm. It is important to note that the replicate variation was lowest when the samples were first mashed (Day 0) and highest at the end of fermentation (Day 5). This suggests that the fermentation process normalized the diverse samples, reducing differences due to variety or terroir between them.

**Table 6:** Effect tests from ANCOVA of specific gravity x hours of fermentation.

Source	Nparm	DF	Sum of Squares	F Ratio	Prob > F
Treatment	9	9	0.00094879	7.8261	< .0001*
Hours	1	1	0.08245726	6121.289	< .0001*
Treatment*Hours	9	9	0.00034847	2.8743	0.0046*

**Table 7:** Percent of total variance for specific gravity measurements of mash and fermentation as determined through REML.

Effect	Day 0 (Mash)	Day 1	Day 3	Day 4	Day 5
Terroir	45.4%	36.7%	54.3%	36.1%	35%
Variety	16.2%	0%	1.4%	4.7%	11.5%
Variety*Terroir	9.5%	9.3%	0%	9.5%	0%
Batch[Variety,Terroir]	28.9%	54%	44.3%	49.7%	53.5%
Sum Total Variance	100%	100%	100%	100%	100%
Units	Specific Gravity	Specific Gravity	Specific Gravity	Specific Gravity	Specific Gravity
Mean Value +/- SE	1.1 +/- 0.0	1.05 +/- 0.0	1.03 +/- 0.0	1.0 +/- 0.0	0.99 +/- 0.0
Observations (n)	30	17	15	27	30

#### Dextrins, sugars, and ethanol

While specific gravity is a rapid and informative measurement, it does not discriminate between the various types of sugars. Further, it does not provide a direct measurement of ethanol concentration. HPLC is able to effectively separate and quantify DP4+ (dextrins), DP3 (maltotriose), maltose, glucose, and ethanol. Table 8 shows the variance components for these compounds at Day 0 and Day 5.

**Table 8:** Percent of total variance for DP4+, DP3, maltose, glucose, and ethanol at Day 0 and Day 5 (with and without outliers removed) of fermentation as determined through REML.

Effect	a) Day 0				
	DP4+	DP3	Maltose	Glucose	Ethanol
Terroir	0%	12.1%	0%	0%	ND
Variety	6%	0%	0%	5%	ND
Variety*Terroir	57%	20.6%	68.6%	8.3%	ND
Batch[Variety,Terroir]	37%	67.3%	31.4%	86.7%	ND
Sum Total Variance	100%	100%	100%	100%	ND
Units	wt/vol	wt/vol	wt/vol	wt/vol	% ABW
Mean Value +/- SE	2.5 +/- 0.2	0.1 +/- 0.0	2.9 +/- 0.2	7.9 +/- 0.2	
Observations (n)	26	26	26	26	26
Effect	b) Day 5 (with outliers)				
	DP4+ (wt/vol)	DP3 (wt/vol)	Maltose (wt/vol)	Glucose (wt/vol)	Ethanol (% ABW)
Terroir	21.6%	0%	38.9%	40.1%	5.5%
Variety	0%	0%	5.1%	10.6%	0%
Variety*Terroir	0%	0%	0	0%	5.5%
Batch[Variety,Terroir]	78.4%	100%	56%	49.3%	89%
Sum Total Variance	0.000212	100%	100%	100%	100%
Units	wt/vol	wt/vol	wt/vol	wt/vol	% ABW
Mean Value +/- SE	0.1 +/- 0.0	0.03 +/- 0.0	0.2 +/- 0.0	0.9 +/- 0.4	6.7 +/- 0.2
Observations (n)	30	30	30	30	30
Effect	c) Day 5 (outliers removed)				
	DP4+ (wt/vol)	DP3 (wt/vol)	Maltose (wt/vol)	Glucose (wt/vol)	Ethanol (% ABW)
Terroir	45.8%	0%	45.9%	36.8%	38.9%
Variety	7.7%	0%	1.2%	10.3%	19.7%
Variety*Terroir	0%	0%	0%	0%	0%
Batch[Variety,Terroir]	46.5%	100%	52.9%	52.9%	41.4%
Sum Total Variance	100%	100%	100%	100%	100%
Units	wt/vol	wt/vol	wt/vol	wt/vol	% ABW
Mean Value +/- SE	0.1 +-/ 0.0	0.03 +/- 0.0	0.2 +/- 0.0	0.9 +/- 0.4	6.8 +/- 0.2
Observations (n)	27	27	27	27	27

(a) Four batch replicates were not included for Day 0 HPLC analysis (Mycogen-Hidalgo Batch 2, Mycogen-Hidalgo Batch 3, Terral-Calhoun Batch 3, and Terral-Hansford Batch 3) due to loss of sample during HPLC analysis. ND = Not Detected. (c) Three Outliers were removed (Mycogen-Calhoun Batch 2, Mycogen-Hill Batch 2, and Terral-Hill Batch 3).

At Day 0, post-mashing, much of the variation in dextrins and maltose was due to the interaction effect. Maltotriose shows a similar result, although to a lesser extent. The residual was responsible for most of the variation in glucose (Table 8).

**Fig 2:**
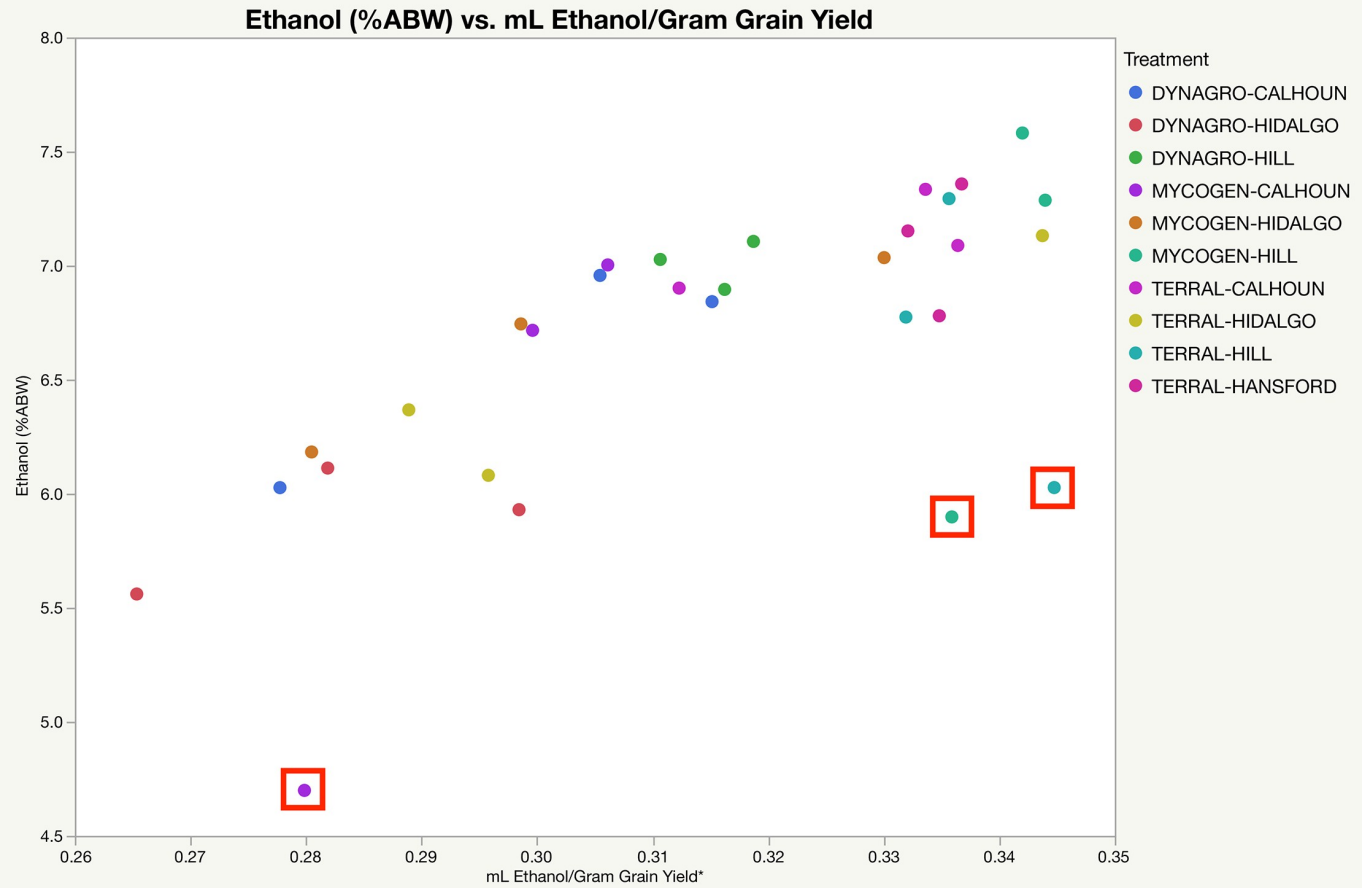
Ethanol % in beer vs. ethanol yielded after distillation. Ethanol (%ABW) was measured in beer post fermentation via HPLC-RID. mL Ethanol/gram of grain was measured by distilling beer into new-make bourbon, collecting identical volumes per batch (550 mL), and measuring density with a density meter. Red boxes denote the three outlier data points that were removed for subsequent REML analysis (Table 8).

It was somewhat surprising that the ethanol concentration at Day 5, post-fermentation, did not show variation due to terroir or variety. As reported later alcohol yield did show variation due to variety and terroir. As explained in Materials & Methods, three outliers (Fig 2) were identified in the data. Removing these outliers (Table 8C), the results showed that terroir and variety were responsible for 39% and 20% of the experimental variation, respectively. This better aligns with the impact of terroir and variety on alcohol yield variation. Further, much of the variation in the concentrations of dextrins, maltotriose, and glucose at Day 5 was due to terroir. The results from mash and fermentation analysis suggest that variety and terroir do impact starting fermentable extract and attenuation, which can impact alcohol yield.

### New-make bourbon analysis

#### Alcohol yield

While starch levels in corn, sugar yields during mashing, and alcohol production during fermentation are important measurements for assessing alcohol yield, distillers ultimately determine yield through measurement after distillation. As is described, each treatment was exposed to identical mashing, fermentation, and distillation procedures. After distillation, the milliliters of ethanol per gram of corn was measured (Table 9). Both terroir and variety were responsible for 32% and 24% of the experimental variation, respectively. The conservative REML best linear unbiased predictions ranged from 0.29 ml ethanol per gram of grain yield (Dyna-Gro, Monte Alto) to 0.34 (Terrel, Sawyer Farms), which would mean 17% more corn would need to be purchased for the same whiskey production.

**Table 9:** Percent of total variance for alcohol yield of new-make bourbon as determined through REML.

Effect	mL EtOH / Gram Corn
Terroir	32.3%
Variety	24.1%
Variety*Terroir	1.2%
Batch[Variety,Terroir]	42.4%
Sum Total Variance	100%
Units	mL
Mean Value +/- SE	0.31 +/- 0.0
Observations (n)	30

Multivariate analsysis (Table 10, Table 11) shows that starch percentage, total extract (the sum of DP4+, DP3, maltose, and glucose), ethanol (%ABW), and the ultimate alcohol yield all possess statistically significant correlations. This is important, as it further supports the notion that variation from variety and terroir across starch percentage in the corn, total extract post-mashing, and ethanol (%ABW) post-fermentation can ultimately impact ethanol yield post-distillation.

**Table 10:** Correlation probabilities (Prob > F) among starch concentration in corn, ethanol (%ABW) post-fermentation, and ethanol yield post-distillation.

	Starch % Dry Basis	Ethanol (%ABW)	mL EtOH/Gram Corn
Starch % Dry Basis	< .0001	0.1751	0.0273
Ethanol (%ABW)	0.1751	< .0001	< .0001
mL Ethanol/Gram Grain Yield	0.0273	< .0001	< .0001

Observations (n) = 60

**Table 11:** Correlation probabilities (Prob > F) among starch concentration in corn, total extract post-mashing (Day 0), ethanol (%ABW) post-fermentation (Day 5), and ethanol yield post-distillation.

	Starch % Dry Basis	Total Extract	Ethanol (%ABW)	mL Ethanol/Gram Grain Yield
Starch % Dry Basis	< .0001	0.0118	0.1926	0.1204
Total Extract	0.0118	< .0001	0.0289	0.0033
Ethanol (%ABW)	0.1926	0.0289	< .0001	0.0003
mL Ethanol/Gram Grain Yield	0.1204	0.0033	0.0003	< .0001
Obersvations (n) = 56				

Four batch replicates were not included for total extract post-mashing (Day 0) HPLC analysis (Mycogen-Hidalgo Batch 2, Mycogen-Hidalgo Batch 3, Terral-Calhoun Batch 3, and Terral-Hansford Batch 3) due to loss of sample during HPLC analysis.

#### Congeners and aromas

Samples of new-make bourbon were exposed to GC-MS/O and descriptive sensory analysis techniques. GC-MS/O detected 68 different compounds (16 more than the milled corn) that registered an aroma event via olfactometric detection by a trained operator. Sensory analysis utilized a trained panel to detect and quantify up to 54 different new-make whiskey aromas. Table 12 and Table 13 below provide the percent of total variance for those congeners and aromas where the residual effect was responsible for no more than ~80% of the variation.

**Table 12:** Percent of total variance for new-make bourbon congener concentrations as determined through REML.

Effect	GC-MS/O								
	Isoamyl acetate	2-methylbutyl decanoate	Ethyl 2-nonenoate	2-tridecanone	2,4-decadienal	Ethyl sorbate	Isopentyl hexanoate	Ethyl acetate	4-vinylanisole
Terroir	9.8%	0%	67.9%	0.9%	29.7%	1.2%	21.8%	24.6%	47.9%
Variety	3.6%	0%	0%	25.6%	0%	2.5%	0.7%	0%	15.1%
Variety*Terroir	23.2%	49.2%	0%	0%	0%	15%	0%	2.8%	16.1%
Batch[Variety,Terroir]	63.4%	50.8%	32.1%	73.5%	70.3%	81.3%	77.5%	72.6%	20.9%
Sum Total Variance	100%	100%	100%	100%	100%	100%	100%	100%	100%
Units	Ion Count	Ion Count	Ion Count	Ion Count	Ion Count	Ion Count	Ion Count	Ion Count	Ion Count
Mean Value +/- SE	189638.3 +/- 56844.9	38039.0 +/- 38039.0	96846.9 +/- 55953.4	30039.3 +/- 20075.5	126750.6 +/- 35235.9	32691.29 +/- 5571.4	30471.4 +/- 17721.7	679113.7 +/- 122738.8	445662.2 +/- 114215.8
Observations (n)	30	30	30	30	30	30	30	30	30
Effect	GC-MS/O								
	Ethyl decanoate	Ethyl dodecanoate	Acetal	Styrene	Ethyl undecanoate	(*E*)-2-heptenal	2-methyl-5-isopropenylfuran	Ethyl (*E*)-2-octenoate	Ethene, ethoxy-
Terroir	43.1%	3.1%	29.4%	6.7%	64.4%	18.1%	0%	49.4	0%
Variety	21.8%	3.6%	17.6%	35.3%	8%	22.4%	20.8%	0%	1.5%
Variety*Terroir	0%	17.1%	0%	1.5%	4.5%	0%	1.7%	0%	35.5%
Batch[Variety,Terroir]	35.1%	76.2%	53%	56.5%	23.1%	59.5%	77.5%	50.6%	63%
Sum Total Variance	100%	100%	100%	100%	100%	100%	100%	100%	100%
Units	Ion Count	Ion Count	Ion Count	Ion Count	Ion Count	Ion Count	Ion Count	Ion Count	Ion Count
Mean Value +/- SE	51696255 +/- 10687333	3899065.2 +/- 1024933	131682.7 +/- 66275.5	1013993.7 +/- 436857.9	183871.0 +/- 83917.6	67719.8 +/- 38377.9	10263.4 +/- 8670.2	196254.5 +/- 79899.0	3746.1 +/- 3654.0
Observations (n)	30	30	30	30	30	30	30	30	30
Effect	GC-MS/O								
	Ethyl trans-4-decenoate	Ethyl heptanoate	Ethyl hexanoate	Napthalene	Nonanal	Ethyl nonanoate	(*E*)-2-nonenal	Isoamyl octanoate	Ethyl octanoate
Terroir	68.6%	21.2%	21.6%	21.8%	6.9%	39.1%	9.6%	34.6%	7.4%
Variety	5.2%	11.5%	10.4%	0%	24.1%	12.2%	18.5%	9.9%	32.2%
Variety*Terroir	0%	0%	0%	10.9%	8.9%	0%	0%	0%	4.3%
Batch[Variety,Terroir]	26.2%	67.3%	68%	67.3%	60.1%	48.7%	71.9%	55.5%	56%
Sum Total Variance	100%	100%	100%	100%	100%	100%	100%	100%	100%
Units	Ion Count	Ion Count	Ion Count	Ion Count	Ion Count	Ion Count	Ion Count	Ion Count	Ion Count
Mean Value +/- SE	2335925.5 +/- 705858.2	275450.2 +/- 83277.6	897135.5	19611.0 +/- 17922.4	350703.97 +/- 118026.4	2551498.7 +/- 743395.8	303987.0 +/- 72030.9	1104644.7 +/- 289180.2	22363759 +/- 6611977
Observations (n)	30	30	30	30	30	30	30	30	30
Effect	GC-MS/O								
	Ethyl hept-2-enoate	2-Nonanone	2-octenal, (E)-	2-undecanone	Phenylethyl alcohol	Acetophenone	2-pentylfuran	Ethyl (E)-4-hexenoate	Cedr-8-ene
Terroir	32.1%	5%	26.2%	23.2%	16.2%	9.1%	37.3%	0%	72.5%
Variety	0%	17.1%	0%	1.4%	0%	0%	8%	19%	0%
Variety*Terroir	4.5%	10%	0%	63.1%	27.5%	42%	0%	28.2%	0%
Batch[Variety,Terroir]	63.4%	67.9%	73.8%	12.3%	56.3%	48.9%	54.7%	52.8%	27.5%
Sum Total Variance	100%	100%	100%	100%	100%	100%	100%	100%	100%
Units	Ion Count	Ion Count	Ion Count	Ion Count	Ion Count	Ion Count	Ion Count	Ion Count	Ion Count
Mean Value +/- SE	52677.9 +/- 20017.3	11597.9 +/- 7730.7	102855.3 +/- 41003.2	19602.0 +/- 20491.3	307577.2 +/- 87401.5	5469.8 +/- 4026.9	117483.8 +/- 61497.9	15337.2 +/- 8193.0	117628.7 +/- 116189.1
Observations (n)	30	30	30	30	30	30	30	30	30

Ion Count **=** Total ion count (TIC) area under the curve and compound identity was based on the NIST library.

**Table 13:** Percent of total variance for new-make bourbon aroma concentrations as determined through REML. Intensity corresponds to an aroma intensity, with a scale of 0–15, and was determined by the trained sensory panel.

Effect	Descriptive Sensory Analysis												
	Alcohol	Sweet	Sour	Grain Complex	Corn	Malt	Woody	Musty Earthy	Molasses	Anise	Lactic Acid	Stale	Prickle Pungent
Terroir	0.%	4.7%	0%	0%	0%	35.9%	8.8%	0%	0%	63%	0.6%	14.4%	5.8%
Variety	25.9%	21.9%	20.8%	26.6%	2.6%	0.4%	34.2%	14.6%	19.6%	0%	0%	0.9%	32.2%
Variety*Terroir	24.4%	0%	12%	0%	42.5%	0%	0%	8%	0%	0%	48.6%	9%	7.2%
Batch[Variety,Terroir]	69.7%	73.4%	67.2%	73.4%	54.9%	63.7%	57%	77.4%	80.4%	37%	50.8%	75.7%	54.8%
Sum Total Variance	100%	100%	100%	100%	100%	100%	100%	3.44E-01	100%	100%	100%	100%	100%
Units	Intensity	Intensity	Intensity	Intensity	Intensity	Intensity	Intensity	Intensity	Intensity	Intensity	Intensity	Intensity	Intensity
Mean Value +/- SE	6.6 +/- 0.2	2.6 +/- 0.2	3.1 +/- 0.3	5.3 +/- 0.2	5.1 +/- 0.2	3.6 +/- 0.2	3.9 +/- 0.3	2.9 +/- 0.2	1.2 +/- 0.3	0.3 +/- 0.2	1.9 +/- 0.2	2.4 +/- 0.1	4.0 +/- 0.2
Observations (n)	30	30	30	30	30	30	30	30	30	30	30	30	30

Intensity corresponds to an aroma intensity, with a scale of 0–15, and was determined by the trained sensory panel.

Of the thirty-six congeners identified in Table 12 where the concentration showed substantial variance beyond the residual (i.e. more than ~20% of the total variance), 50% were esters, 14% were aldehydes, and 11% were ketones. The fact that such a large percentage of esters displayed variation due to variety and terroir is encouraging, as esters are also important flavor contributors in new-make whiskey, usually contributing fruity characteristics. Aldehydes and ketones are also important flavor contributors, providing fruity, floral, grassy, and fatty aromas.

Thirteen aromas were detected via Spectrum sensory analysis where the residual effect was not responsible for more than ~80% of the total variance component (Table 13). Variance was largely due to terroir in three (Malt, Anise, Stale), variety in seven (Sweet, Sour, Grain Complex, Woody, Musty Earthy, Molasses, Prickle Pungent), and the interaction of terroir and variety in three (Alcohol, Corn, Lactic Acid).

Many of the congeners identified in Table 12 have been reported previously as being important contributors to flavor in bourbon. Poisson and Schieberle utilized aroma extract dilution analysis (AEDA), quantitative measurements, aroma recombination, and omission studies to identify the most odor-active congeners in whiskey [51,52]. From the compounds they identified, the following were also identified in this report (Table 12), grouped according to compound class: *esters*—isoamyl acetate, ethyl acetate, ethyl hexanoate, ethyl octanoate; *aldehydes*—(*E*)-2-heptenal, nonanal, (*E*)-2-nonenal, 2,4-decadienal; *fusel alcohol*—phenylethyl alcohol; and acetal. According to Poisson and Schieberle, the esters listed contribute fruity flavors. The aldehydes (*E*)-2-heptenal, (*E*)-2-nonenal, and 2,4-decadienal contribute fatty and green flavors, and nonanal contributes soapy flavors. Phenylethyl alcohol is known for imparting rose and floral aromas. Acetal (also called 1,1-diethoxyethane) contributes fruity and ethereal flavors.

The only congener found in both milled corn and new-make from Table 4 and Table 12 where the respective concentrations showed substantial variance beyond the residual was ethyl decanoate. Ethyl decanoate has previously been identified in bourbon [61], described as having a fruity, apple aroma. Importantly, combined among all new-make samples, ethyl decanoate had the highest peak area value (averaged 48 million across samples, more than double the next highest ethyl octanoate with 22 million) out of the sixty-eight congeners detected. Combined among all corn samples, ethyl decanoate had the ninth highest peak area value (averaged 60,421 across samples, hexanal was the highest with 280,095) out of fifty-two congeners detected. However, ethyl decanoate concentration in corn did not show significant correlation to ethyl decanoate concentrationin in new-make. This might suggest that ethyl decanoate present in corn might be concentrated, created or altered during the mashing, fermentation, or distillation processes of whiskey production. Another possibility is that yeast production of deacoic acid and/or ethyl decanoate is impacted by other compositional aspects of corn, and these aspects can negate varying contributions from the corn kernels themselves.

While the presence and concentration of certain congeners can correlate with aroma flavors and concentrations, this is not always the case. As pointed out by Poisson and Schieberle, more than 300 compounds have been identified in whiskey, yet only a subset of these (likely 30 to 60) are important for flavor. Therefore, we aimed to determine if there were any important correlations between congeners and aroma in new-make. First, looking at each relationship between congeners and aroma individually, moderate to no correlations were found in most cases and nothing was identified that warrented discussion. However, instead of considering each aroma individually, we grouped them into two categories, denoted as “good” and “bad” aromas and summed the individual aroma concentrations generated by the Spectrum method. These good and bad catagories corresponded to aromas that are typically deemed desirable and undesirable (S10 Table), respectively, in new-make bourbon. Further, we considered all detected congeners and aromas, not just those reported in Table 12 and Table 13.

Of the 68 new-make bourbon congeners identified by GC-MS/O, seven were found to possess both statistical (i.e. p-values) and practical (i.e. effect sizes) significance with the summed value Total Aroma Units—Good (Table 14). Four of these seven congeners were esters (isoamyl acetate, ethyl nonanoate, ethyl octanoate, and ethyl (*E*)-4-hexenoate), known to impart desirable fruity flavors to whiskey. Nonanal imparts soapy characteristics, which is typically deemed a desirable aroma contributor to a whiskey’s flavor. Acetaldeyhyde was the only congener that showed a negative correlation to Total Aroma Units—Good. This is not surprising, as high-levels of acetaldehyde impart astringent, solventy, and green apple flavors. The majority of this compound is typically discarded during distillation, at the discretion of the distiller’s judgment. Styrene is usually attributed to phenolic and plastic flavors. While in isolation these flavors are negative, a certain level for phenolic nuances are usually desired in whiskey.

**Table 14:** Significant correlation probabilities (Prob > F) of new-make bourbon congeners with Total Aroma Units—Good and Total Aroma Units—Bad.

	Isoamyl acetate	Acetaldehyde	Nonanal	Ethyl Nonanoate	Ethyl Octanoate	Styrene	Ethyl (*E*)-4-hexenoate	(*E*)-2-nonenal
Total Aroma Units—Good	0.3352*	-0.3846**	0.3734**	0.3182*	0.4270**	0.4542**	0.5067***	0.2991^NS^
Total Aroma Units—Bad	0.1558^NS^	0.0309^NS^	0.1100^NS^	0.2194^NS^	0.2282^NS^	0.1019^NS^	0.0061^NS^	0.4669***

n = 30

Values reported are strength and direction of correlation (R).

*, **, *** indicate statistical significance at the 10%, 5%, and 1% level respectively. Given the limited sample size (n = 30), we consider the 10% level to be practically useful and significant. NS indicate non-significant differences.

Likewise, of the 68 new-make bourbon congeners identified by GC-MS/O, only (*E*)-2-nonenal was found to possess both statistical and practical significance with the summed value Total Aroma Units—Bad (Table 14). Given that it is known to harbor aromas of cardboard, staleness, and body odor [62], it is not surprising that increased levels of (*E*)-2-nonenal led to undesirable aromas in the new-make bourbon samples considered here.

All of the bourbon new-make congeners listed in Table 14 were also highlighted in Table 12, with their concentrations showing substantial variance beyond the residual. This indicates that certain new-make bourbon congeners that are significantly correlated with overall desireable and undesirable flavors in new-make bourbon also show concentration variations due to variety and terroir. Further, of the eight bourbon new-make congeners listed in Table 14, only three of them (ethyl nonanoate, styrene, and ethyl (*E*)-4-hexenoate) were not listed by Poisson & Schieberle as being important contributors in bourbon.

Next, we aimed to determine if any milled corn congeners correlated with the new-make bourbon congeners identified in Table 14. We found benzaldehyde concentration in milled corn correlated with both statistical and practical significance to isoamyl acetate (R = 0.5148***), nonanal (R = 0.4790***), styrene (R = 0.4221**), and ethyl octanoate (R = 0.5042***) concentrations in new-make bourbon. Ethyl octanoate is especially interesting, due to it’s strong correlation to Total Aroma Units—Good in new-make bourbon. Further, among the 68 congeners identified in new-make bourbon, ethyl octanoate had the second highest total peak area value for the sum of all measured samples (n = 30). In general, benzaldehyde concentration in corn statistically and practically correlated with a number of other congener concentrations in new-make bourbon, such as: isoamyl alcohol (R = 0.3850**), Benzene, 1-ethenyl-4-methoxy (R = 0.4494**), ethyl-trans-4-decenoate (R = -0.4261**), and phenylethyl alcohol (R = 0.4380**), some of which were noted in Table 12 and highlighted by Poisson and Schieberle to be important controbutors to flavor in bourbon. Importantly, benzaldehyde concentration in corn kernel did not correlate with (*E*)-2-nonenal concentation in new-make bourbon.

Benzaldehyde concentration in milled corn was, however, not significantly correlated with Total Aroma Units—Good in new-make bourbon (R = 0.2837^NS^). However, once a single outlier was removed, the correlation improved (R = 0.3620*). Given that benzaldehyde concentration in corn is greatly influenced by variety and terroir (Table 4), and is readily measured by GC-MS without the expense of creating new-make or conducting sensory analysis, it might have practicle use in selecting improved corn for whiskey.

**Fig 3:**
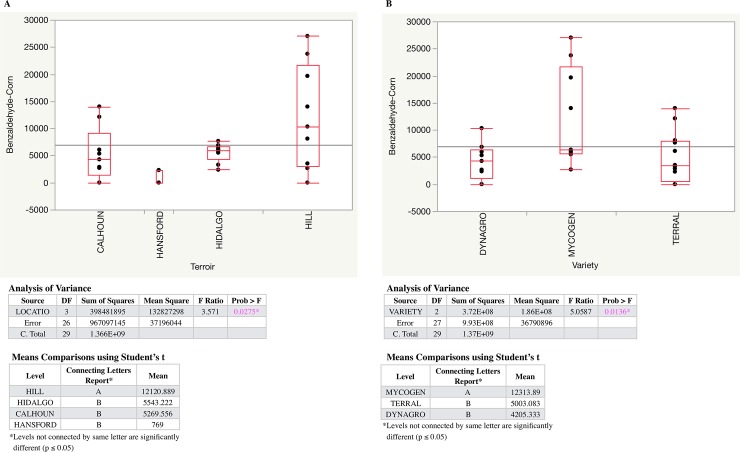
ANOVA and mean comparisons of benzaldehyde concentrations in corn. The dependent variable is peak area. (A) Individual peak areas for each terroir, accompanied with ANOVA and mean comparison analyses. (B) Individual peak areas for each variety, accompanied with ANOVA and mean comparison analyses.

In recent years, it has become common for chefs, bakers, maltsters, and brewers to collaborate with plant breeders in an effort to breed and select for crop varieties that deliver new or forgotten flavors [63,64,65,66]. It is our belief that breeding and better selecting corn growing locations for specific compounds, such as increased benzaldehyde concentrations, has potential to deliver improved corn that possess heightened and desirable flavors in new-make bourbon. In this study, 2C797—Mycogen Seed benzaldehyde concentration was found to be significantly higher than the other two varieties (Fig 3B). Here we found corn from the Hill county terroir contained significantly higher concentrations of benzaldehyde than the other three terroirs (Fig 3A).

**Fig 4:**
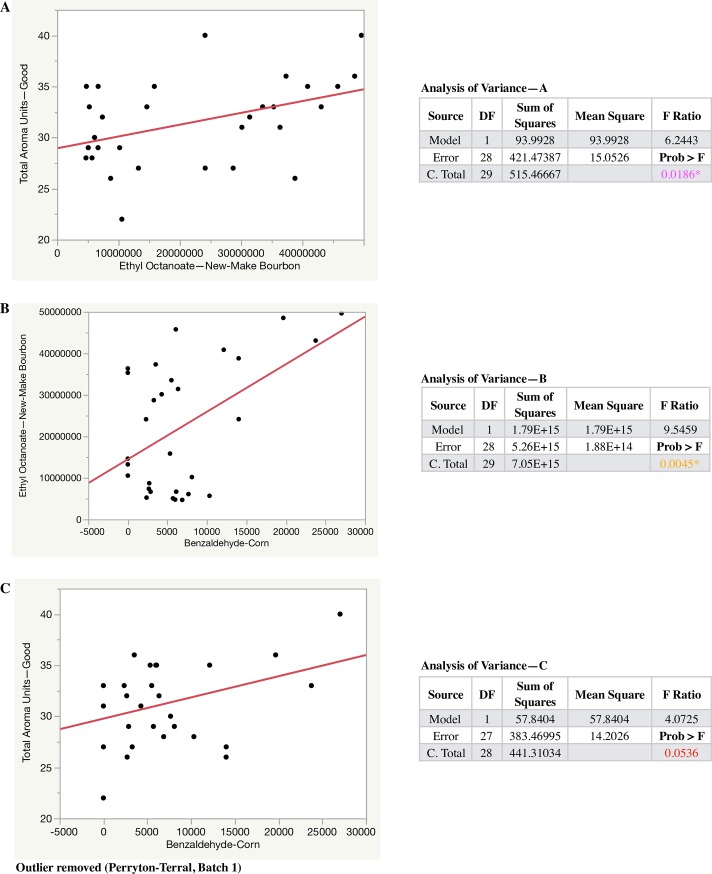
Benzaldehyde in corn and ethyl octanoate new-make bourbon are linked to desirable aromas in new-make. (A) Ethyl octanoate concentration in new-make is positively correlated with Total Aroma Units—Good concentration. (B) Benzaldehyde concentration in corn is positively correlated with ethyl octanoate concentration in new-make bourbon. (C) Benzaldehyde concentration in corn is positively correlated with Total Aroma Units—Good concentration.

To show the progression of analyses that elucidate how desirable aromas in new-make bourbon can be linked to congeners in new-make bourbon and corn, Fig 4. Shows the indivudal linear regressions and ANOVA results.

## Conclusions

To our knowledge, this is the first report to investigate the impact of variety and terroir on flavor and alcohol yield in new-make bourbon. Our findings suggest that even among commodity yellow dent corn hybrid varieties, there variations in flavor and alcohol yield potential still occur and can be targeted. Further, we showed that the different Texas terroirs impacted both flavor and alcohol yield. Lastly, our results suggests that benzaldehyde might be an important chemical marker in corn for plant breeders and distillers to consider, as it is linked to the increase of desirable congeners and aromas in new-make bourbon. Based on these findings, if distillers are searching for improved flavor and yield via corn, or if they simply wish to maintain greater consistency in their whiskey, then both variety and terroir need to be considered.

In the future it will be important to confirm that flavor differences in new-make are maintained during and after oak barrel maturation. Experiences from a large two-variety batch study suggest that flavor nuances due to variety and terroir are not masked during or after maturation in oak, and in fact may be greater, but this will need to be confirmed in future replicated trials. Lastly, while this study found terroir to, in general, have a greater impact on variation, only commodity yellow dent corn hybrids were investigated. Commodity hybrid varieties contain limited genetic diversity as compared to the varieties that exist as heirlooms, open pollinated varieties, and tropical hybrids. Such a study is currently underway by the authors.

## Supporting information

S1 Table Raw data behing proximate analysis of whole and milled corn. (XLSX)

S2 Table Raw data behind GC-MS/O analysis of milled corn. (XLSX)

S3 Table Raw data behind Spectrum sensory analysis of milled corn. (XLSX)

S4 Table Raw data behind specific gravity and pH analysis of fermentations. (XLSX)

S5 Table Raw data behind HPLC analysis of Post Mashing and Post Fermentation. (XLSX)

S6 Table Raw data behind alcohol yield analysis. (XLSX)

S7 Table Raw data of new-make bourbon proof after spirit run distillation. (XLSX)

S8 Table Raw data behind GC-MS/O analysis of new-make bourbon. (XLSX)

S9 Table Raw data behind Spectrum sensory analysis of new-make bourbon. (XLSX)

S10 Table Segregation of GOOD and BAD aromas in new-make bourbon. (XLSX)
